# Characterization of the adult *Aedes aegypti* early midgut peritrophic matrix proteome using LC-MS

**DOI:** 10.1371/journal.pone.0194734

**Published:** 2018-03-23

**Authors:** Shavonn R. Whiten, W. Keith Ray, Richard F. Helm, Zach N. Adelman

**Affiliations:** 1 Department of Entomology, Texas A&M University, College Station, Texas, United States of America; 2 Department of Biochemistry, Virginia Polytechnic Institute and State University, Blacksburg, Virginia, United States of America; Yale School of Public Health, UNITED STATES

## Abstract

The *Aedes aegypti* mosquito is the principal vector of arboviruses such as dengue, chikungunya, yellow fever, and Zika virus. These arboviruses are transmitted during adult female mosquito bloodfeeding. While these viruses must transverse the midgut to replicate, the blood meal must also reach the midgut to be digested, absorbed, or excreted, as aggregation of blood meal metabolites can be toxic to the female mosquito midgut. The midgut peritrophic matrix (PM), a semipermeable extracellular layer comprised of chitin fibrils, glycoproteins, and proteoglycans, is one such mechanism of protection for the mosquito midgut. However, this structure has not been characterized for adult female *Ae*. *aegypti*. We conducted a mass spectrometry based proteomic analysis to identify proteins that comprise or are associated with the adult female *Ae*. *aegypti* early midgut PM. Altogether, 474 unique proteins were identified, with 115 predicted as secreted. GO-term enrichment analysis revealed an abundance of serine-type proteases and several known and novel intestinal mucins. In addition, approximately 10% of the peptides identified corresponded to known salivary proteins, indicating *Ae*. *aegypti* mosquitoes extensively swallow their own salivary secretions. However, the physiological relevance of this remains unclear, and further studies are needed to determine PM proteins integral for midgut protection from blood meal derived toxicity and pathogen protection. Finally, we describe substantial discordance between previously described transcriptionally changes observed in the midgut in response to a bloodmeal and the presence of the corresponding protein in the PM. Data are available via ProteomeXchange with identifier PXD007627.

## Introduction

The *Aedes aegypti* mosquito is the principle vector of arboviruses throughout the tropics and subtropics worldwide [1]. In these regions, *Ae*. *aegypti* transmit dengue, chikungunya, yellow fever, and Zika viruses to humans, resulting in substantial morbidity and mortality worldwide [1–3]. Dengue is the most important mosquito-borne viral disease with more than 200 million reported cases per year [4]. Dengue has been reported in over 90 countries, and alarmingly, dengue incidences have increased more than 30-fold in the last 40 years [5]. Most importantly, dengue has recently been reported in nonendemic areas of the world such as the United States, Southern Europe, and Australia [5].

When adult female mosquitoes take a blood meal, they consume two to three times their normal body weight [6]. Digestion of the blood meal releases a large amount of free heme into the midgut lumen [7, 8]. Free heme can result in the oxidation of nucleic acids [9], lipids [10, 11], and proteins [12, 13]. Therefore, understanding the physiological mechanisms adult female mosquitoes use to process the potentially toxic blood meal and metabolites is needed. In particular, the midgut peritrophic matrix (PM) may serve as protective lining that separates the single cell-layered midgut epithelium from pathogens, abrasion and toxic compounds [7, 14, 15].

The type I PM of the adult mosquito is comprised of proteins, proteoglycans and chitin fibrils [8, 16]. PM proteins, commonly referred to as peritrophins are characterized by the presence of a secretory signal peptide, multiple chitin-binding domains containing cysteine-proline dipeptides and intervening mucin-like domains rich in proline, serine and threonine [17]. The multiple chitin-binding domains of PM peritrophins function as cross-linkers for chitin fibrils, thereby providing structure and support for the PM [18, 19]. Two-dimensional polyacrylamide gel electrophoresis and lectin-binding assays suggest the adult female *Ae*. *aegypti* PM may contain 20–40 major proteins [20]. However, only two proteins have been identified and characterized as adult *Ae*. *aegypti* peritrophins: intestinal mucin 1 (*Ae*IMUC1) [21] and adult peritrophin 50 (AeAper50) [22]. Therefore, we conducted a mass spectrometry based proteomic analysis to obtain a comprehensive understanding of the adult female *Ae*. *aegypti* early PM, with particular interest in adult female PM proteins containing structural features characteristic of peritrophins. Our efforts resulted in the identification of more than 6000 peptides derived from the early PM, corresponding to 474 unique proteins, 115 of which are predicted to be secreted. We identified additional peritrophins and confirm that a substantial number of salivary proteins are delivered to the midgut and may potentially assist in blood meal detoxification and/or digestion.

## Materials and methods

### Mosquito rearing

*Aedes aegypti* (Liverpool strain) mosquitoes were reared under standard insectary conditions at 28 ^o^C and 60–70% relative humidity with a 14:10 light:dark photoperiod. Adult mosquitoes were provided 10% sucrose solution and water. Mosquitoes were starved of 10% sucrose 12 hours prior to experiments.

### Preparation of peritrophic matrix samples for analysis

**Fig 1:**
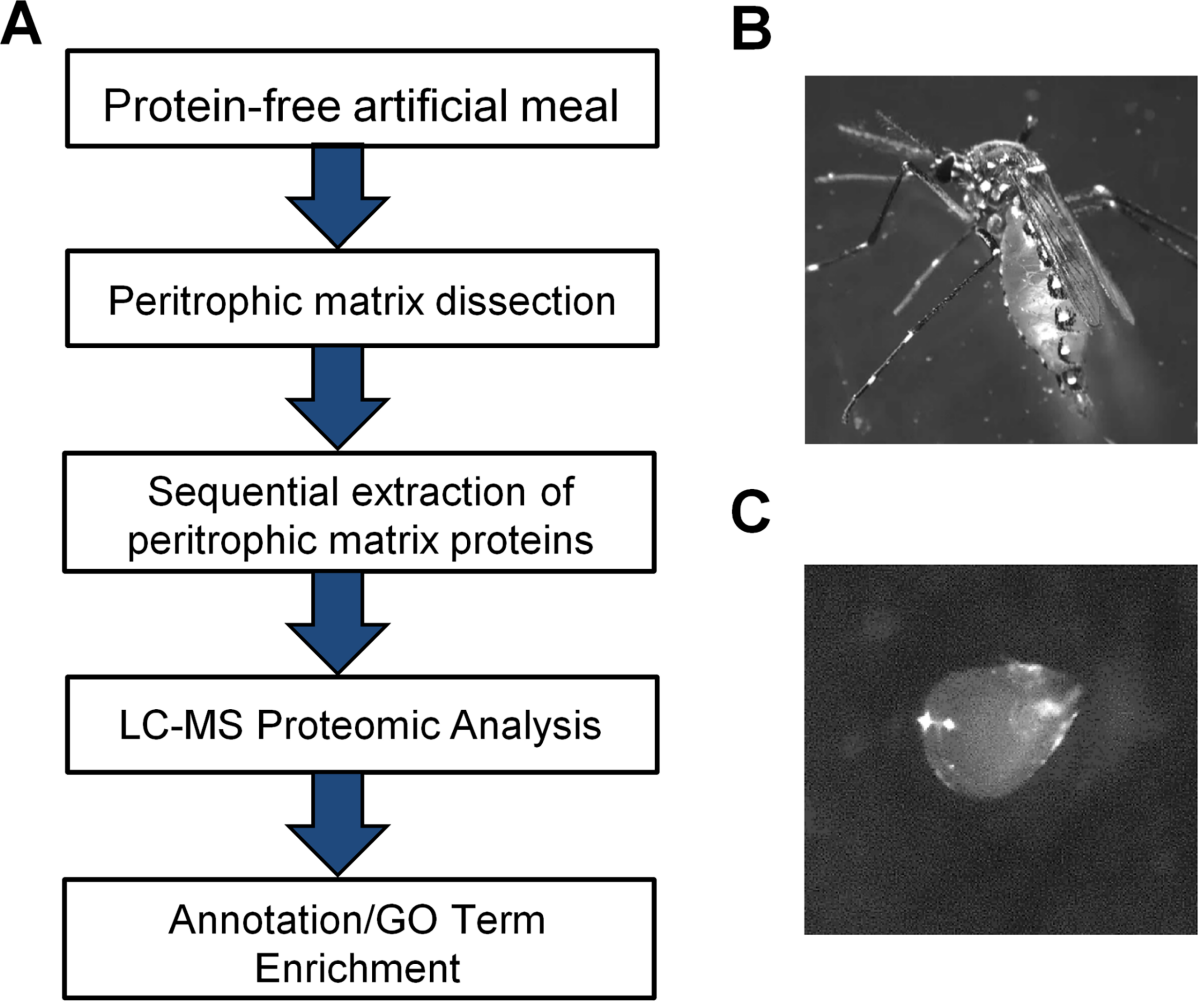
Analysis of *Aedes aegypti* peritrophic matrix proteins. (**A**) Experimental flowchart detailing the feeding of 3–5 day old adult female *Ae*. *aegypti* with protein-free artificial meal containing low melting agarose to produce a rigid PM. Proteomic analysis was conducted (LC-MS). (**B**) Adult female post feeding with protein-free artificial meal containing low melting agarose. (**C**) Six hours post feeding with protein-free artificial meal containing low melting agarose, PMs were dissected in 50% PBS and 50% ethanol solution.

PMs were dissected from 3–5 day old adult female *Ae*. *aegypti* that were fed a protein-free artificial meal (Fig 1). The protein-free artificial meal consisted of 150 mM NaCl, 20 mM NaHCO_3_, and 20 mM ATP as a phagostimulant [20, 23–25]. The protein-free artificial meal also contained 0.2% low melting agarose to provide bulk and induce distension of the midgut [25]. Six hours post-feeding [20], the mosquitoes were immobilized and PMs dissected in 50% EtOH and 50% PBS solution [25]. The dissected PMs were transferred to a 1.5 ml Eppendorf tube and snap-frozen with liquid nitrogen. All 1.5 ml Eppendorf tubes were stored at -80 ^o^C until a total of 1,020 PMs were collected. The 1.5 ml Eppendorf tubes containing PMs were removed from -80^°^C and placed in liquid nitrogen. Extraction buffer (50 mM Tris-HCl, pH 8.5) was added to the first tube and sample homogenized with a plastic pestle. The homogenized sample was transferred to the next tube and PM samples were homogenized. This was repeated for all subsequent tubes until all homogenized samples (800 *μ*l) were combined in a single tube. The pipette tips and plastic pestle were also washed with the extraction buffer. This was referred to as the “Wash” sample. To each of the final tubes, 5 *μ*l of Benzonase was added to remove excess DNA or RNA and then samples were vortexed. Proteins from the 1,020 PMs were sequentially extracted in 200 *μ*l buffer containing either Buffer A (50 mM Tris-HCl, pH 8.5), Buffer B (Buffer A+ 0.5% Triton X-100), or Buffer C (Buffer A+ 2% SDS). The initial Tris fraction and Tris “Wash” fraction were quantified by Bradford assay (0.25 *μ*g/*μ*l and 0.09 *μ*g/*μ*l, respectively). The samples were vortexed, incubated on ice for 1.5 hrs, and then centrifuged in a microcentrifuge at 12,000 x g for 15 minutes. The supernatant containing the extracted proteins was transferred to individual 1.5 Eppendorf tubes (tubes were washed with acetyl nitrile and pre-chilled). For sequential extractions, either Buffer B or Buffer C was added to the pellet fraction and the above extraction procedure was repeated.

### Mass spectrometry based proteomic analysis

The adult female *Ae*. *aegypti* PM proteins were sequentially extracted with i) Buffer A (“Tris” fraction); ii) Buffer B (“Tris-Triton” fraction); iii) Buffer C (“Tris-SDS” fraction). The leftover pellets were washed twice with an excess of Buffer A to remove any excess detergent. This “Pellet” fraction was then analyzed, but no unique proteins were identified. Therefore, this fraction was not considered further. The fractions (“Tris”, “Tris-Triton”, and “Tris-SDS”) were then prepared for digestion with trypsin.

The “Tris”, “Tris-Triton”, and “Tris-SDS” fractions and their respective “Wash” samples were TCA precipitated, and proteins solubilized in 30 *μ*L of 100 mM Tris-HCl, pH 8.5, 8 M urea, reduced with 5 mM TCEP (Tris(2-carboxylethyl)phosphine hydrochloride, Pierce), alkylated with 10 mM IAM (iodoacetamide, Sigma), and deactivated with 10 mM dithiothreitol (DTT). Samples were diluted to 4 M urea with 100 mM Tris-HCl, pH 8.5. Endoproteinase Lys-C (Roche) was added to 0.5 *μ*g [26], and samples were incubated overnight at 37 ^o^C. The next morning, samples were diluted to 1.5 M urea with 100 mM Tris-HCl, pH 8.5. Calcium chloride was added to 2 mM and samples digested with trypsin overnight at 37°C while shaking. The digestion was quenched by adding formic acid to a final concentration of 5%.

The insoluble pellets (“pellet” fractions) from the PM sample and “wash” sample were dried under vacuum, solubilized in 100 *μ*l cyanogen bromide at 500 mg/ml in 88% formic acid, and left in fume hood overnight in the dark [26]. The samples were neutralized by adding 30% ammonium hydroxide drop by drop. To adjust the pH to 8.5, 1M Tris-HCl was added to 100 mM. The samples were then denatured with 8 M urea, reduced with 5 mM TCEP, alkylated with 20 mM IAM, deactivated with 20 mM DTT (1,4-dithiothreitol). The samples were then digested with endoproteinase Lys-C and trypsin as described above. All peptides were cleaned-up using Agilent Bond Elute OMIX pipette tips.

Ten microliters of each resolubilized peptide sample was separated using an Acquity I-class UPLC system (Waters). The mobile phases were solvent A (0.1% (v/v) formic acid (Sigma) in LC/MS grade water (Spectrum Chemicals) and solvent B (0.1% (v/v) formic acid (Sigma) in LC/MS grade acetonitrile (Spectrum Chemicals). The separation was performed using a CSH130 C18 1.7 μm, 1.0 x 150 mm column (Waters) at 50 μL/min using a 110-minute gradient from 3–40% solvent B. The column temperature was maintained at 45°C.

Column effluent was analyzed using a Synapt G2-S mass spectrometer (Waters) using an HDMS^E^ (high-definition mass spectrometry with alternating scans utilizing low and elevated collision energies) acquisition method in continuum positive ion “resolution” MS mode. Source conditions were as follows: capillary voltage, 2.9 kV; source temperature, 125°C; sampling cone, 40 V; desolvation temperature, 350°C; cone gas flow, 50 l/hr; desolvation gas flow, 500 l/hr; and nebulizer gas, 6 bar. Both low energy (no collision energy in either the trap or transfer region) and elevated energy (no collision energy in the trap region and the collision energy ramped based on the bin number exiting the ion mobility cell in the transfer region, see below and Ref. 1) scans were 0.8 seconds each for the m/z range of 100 to 1800. For ion mobility separation, the IMS wave velocity was ramped from 800 to 500 m/sec over the full IMS cycle and the IMS wave height was 40 V. Wave velocity and height in the trap region were 313 m/s and 8 V. Wave velocity and height in the transfer region were 190 m/sec and 4 V. Mobility trapping utilized auto release and mobility separation was delayed 450 μs after trapping.

Collision energy in the transfer region was dependent upon the drift time (bin) within the ion mobility cell as described by Distler et al. (2014) [27]. The CE was ramped from 16–23 V for bins 1 to 40, from 24–47 V for bins 41 to 120, and from 48–60 V for bins 121 to 200.

For lock-mass correction, a 1.2 second low energy scan was acquired every 30 seconds of a 100 fmol/μl [Glu1]-fibrinopeptide B (Waters) solution (50:50 acetonitrile: water supplemented with 0.1% formic acid) infused at 5 μl/min introduced into the mass spectrometer through a different source which was maintained at a capillary voltage of 3.0 kV. The data for lock-mass correction was collected but not applied to sample data until data processing.

Mass spectrometric data from each chromatographic run were processed and analyzed utilizing ProteinLynx Global Server version 3.0.2 (Waters). The software automatically determined average chromatographic and mass spectrometric peak width resolution. Mass values were lock-mass corrected based on the exact m/z value of the +2 charge state of [Glu1]-fibrinopeptide B (785.842). Peaks were defined based on the low energy, elevated energy and bin intensity thresholds of 100, 15 and 750 counts, respectively. The MS and MSMS tolerances for the peptide searches were 24 ppm and 24 ppm, respectively (automatically determined by the Waters PLGS search engine). The final peak list for each sample was then searched against a protein database containing the complete *Ae*. *aegypti* proteome downloaded from VectorBase and 3 randomized decoy entries for each real entry appended using PLGS. Workflow parameters for the protein identification searches were 2 possible missed cleavages utilizing Lys-C and trypsin as the protease combination, a fixed modification of carbamidomethylation of cysteine, possible modifications of glutamine to pyroglutamate when glutamine is present at the N-terminus of a peptide and oxidation of methionine. The software automatically determined peptide and peptide fragment mass tolerances. Protein identification searches using PLGS had a false discovery rate of no more than 5%.

Results for two technical replicates were tabulated utilizing IsoQuant [28]. The final data summary lists only proteins identified by at least 2 unique peptides at a false discovery rate (FDR) less than 3% in both replicates. Proteins were quantified using the Top3 method. Peptides with a minimum replication rate of 2 and a minimum score of 2 per EMRT (exact mass and retention time) cluster, including in-source fragments and those exhibiting a neutral loss of either water or ammonia, were considered valid for protein identification. Only peptides identified as either unique or razor were used for protein quantitation. The mass spectrometry proteomics data have been deposited to the ProteomeXchange Consortium via the PRIDE [29] partner repository with the dataset identifier PXD007627.

### Bioinformatic analyses

The VectorBase Biomart tool was used to obtain predicted structural features for the 474 unique proteins that resulted from our proteomic analysis (AaegL3.3). Previously published RNA-seq transcriptomics data comparing sugarfed and bloodfed adult female *Ae*. *aegypti* [30] was accessed and downloaded to determine proteins isolated in our proteomic analysis with transcripts upregulated five hours after a blood meal. We compared our list of 474 VectorBase Gene Stable IDs for the proteins identified by LC-MS to transcripts found in blood fed females only and transcripts upregulated in bloodfed or sugarfed females.

g: GOSt—gene group functional profiling (version: r1709_e87_eg34) was used to conduct a hypergeometric enrichment analysis for the 115 secreted proteins isolated in our LC-MS based proteomic analysis with a predicted secretory signal peptide [31, 32]. The unordered list of VectorBase Gene Stable IDs was entered in the g: GOSt user interface. Parameters used for the enrichment analysis included: 1) significant values only and 2) hierarchical sorting. The enriched data was output in an Excel spreadsheet (XLSX), and further custom sorted based on p-value. Only results with p-value <0.001 were considered significant.

Secreted midgut proteins were searched for repetitive cysteine-proline dipeptides which are a hallmark characteristic of PM peritophins [7]. The results were then entered into UniProt database and Center for Biological Sequence Analysis (CBS) prediction services database to determine secreted midgut proteins with: 1) two or more chitin-binding domains and 2) *O*-glycosylation and *N*-glycosylation status.

The adult female *Anopheles gambiae* PM proteome list was accesses and downloaded [33] and the VectorBase Gene Stable IDs for the 209 *Anopheles gambiae* PM proteins were entered in VectorBase Biomart. The data was output as a comma separated value (csv) file. We then compared this data with our list of 474 VectorBase Gene Stable IDs. Only one to one orthologs for the two species were kept for further analyses.

## Results

### Proteomic analysis

**Fig 2:**
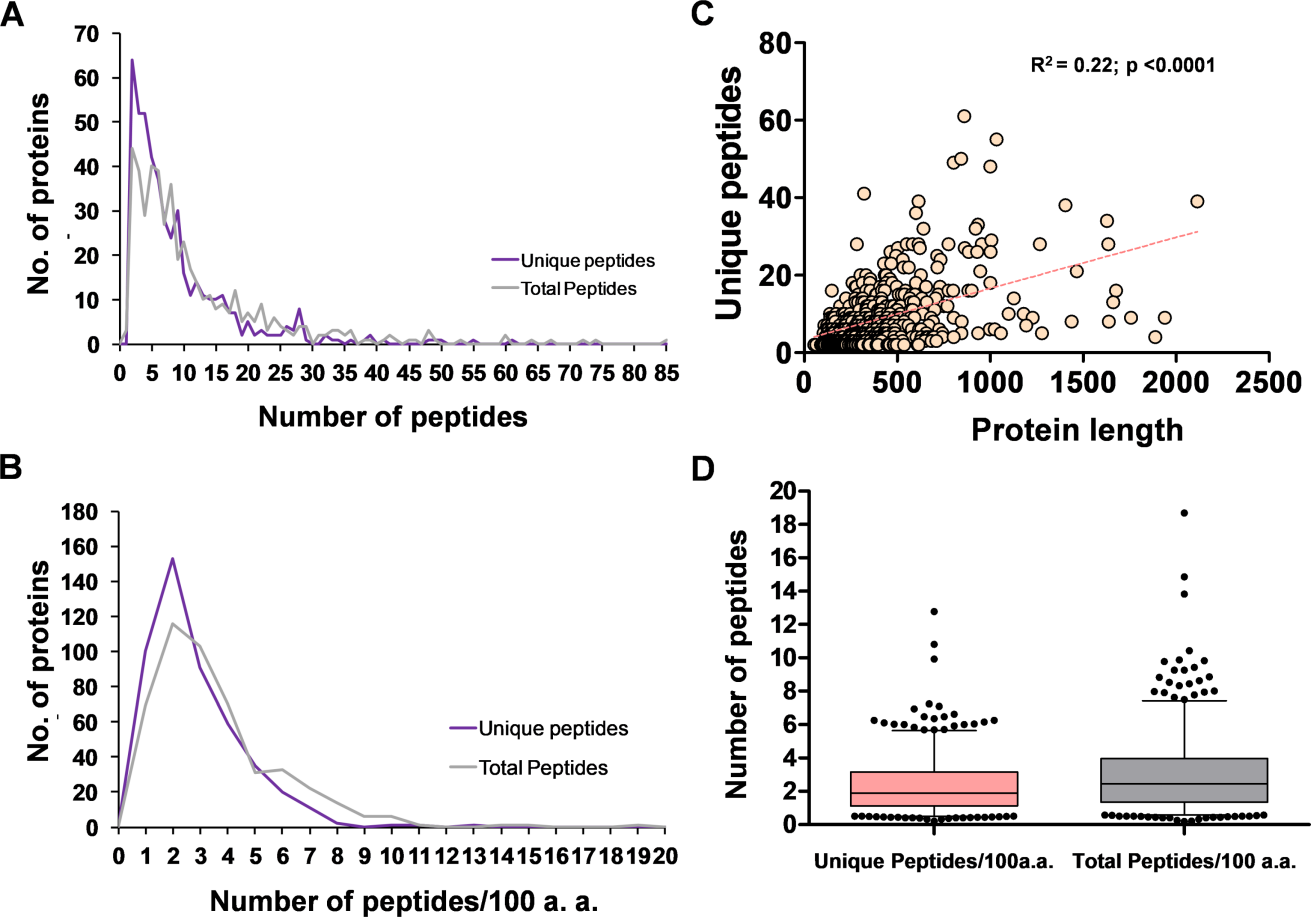
Frequency distribution of the number of peptides recovered per protein. Frequency distribution of the number (No.) of recovered peptides per protein (**A**) or normalized for protein length (**B**). (**C**) Relationship between the predicted length of each protein (Vectorbase AaegL3.3) and the number of unique peptides recovered; dotted line represents the linear regression. (**D**) Box and whisker plot of the number of peptides recovered per protein. Boxes represent the middle quartiles, errors bars the 95% confidence intervals.

Following a protein-free artificial bloodmeal, *Ae*. *aegypti* peritrophic matrices were dissected and proteins were sequentially extracted according to solubility in buffers and detergents (Fig 1). The extracted proteins were then prepared for LC-MS analysis, resulting in the identification of 6292 peptide sequences (4300 unique peptides) corresponding to 474 unique proteins identified with two or more peptides (S1 Table). Given the parent database searched was annotated to encode 15,796 possible proteins, this amounts to exactly 3% of the known protein-coding capacity of *Ae*. *aegypti* being potentially associated with the PM. Recovered peptides ranged from 6–52 amino acids in length, with a mean of 14.4 and a mode of 12 (S1 Fig). Each protein was identified with between 2–61 unique peptides (defined as having distinct coordinates on the matched protein), with a mean/median of 9.1/6.0 peptides per identified protein (Fig 2A). As we observed a weak but significant (and unsurprising) correlation between predicted protein length and the number of unique peptides obtained (Fig 2B), we also calculated the number of peptides obtained per 100 amino acids of protein (Fig 2C). When normalized for protein length, we recovered an average of 3.1 total peptides and 2.3 unique peptides per protein per 100 amino acids (Fig 2D).

**Fig 3:**
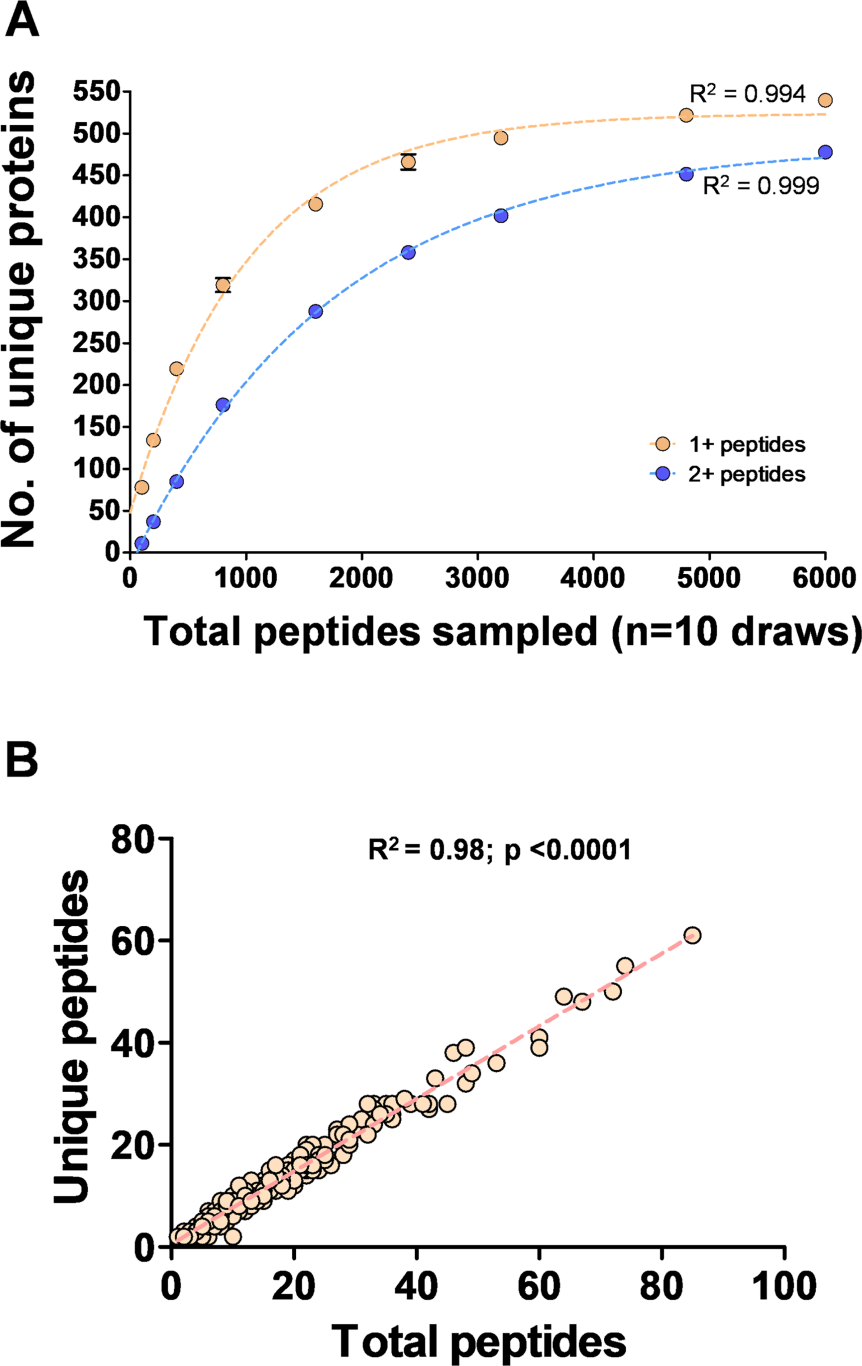
Proteomic analysis of the *Aedes aegypti* peritrophic matrix and contents. (**A**) Each point represents the mean number (No.) of unique proteins identified (with 1+ or 2+ peptides) after 10 random draws from the total dataset of the indicated magnitude. Curves were fit to a one-phase association curve using Graphpad Prism v5.04. (**B**) Linear regression between the total number of peptides recovered and the number of unique peptides.

To assess the completeness of our dataset, we computationally re-sampled an increasing number of random peptides from the full dataset and calculated how many unique proteins were represented. As shown in Fig 3A, the number of unique proteins identified in our dataset is essentially at saturation for unique proteins identified by 1 or more, or 2 or more peptides. Based on these curves, the number of unique proteins that could be identified with 2+ peptides is expected to plateau between 483–491 (95% confidence intervals), indicating we have recovered 96.5–98.1% of the proteins present in the adult PM ~6 hrs after an artificial meal. In contrast, the number of unique peptides per protein had a strong linear relationship with the total number of peptides per protein, indicating our dataset did not reach saturation at the level of coverage of individual proteins (Fig 3B). Taken together, we conclude that identifying more peptides using this methodology is very unlikely to uncover new proteins, but very likely to uncover additional unique peptides from proteins already identified.

**Fig 4:**
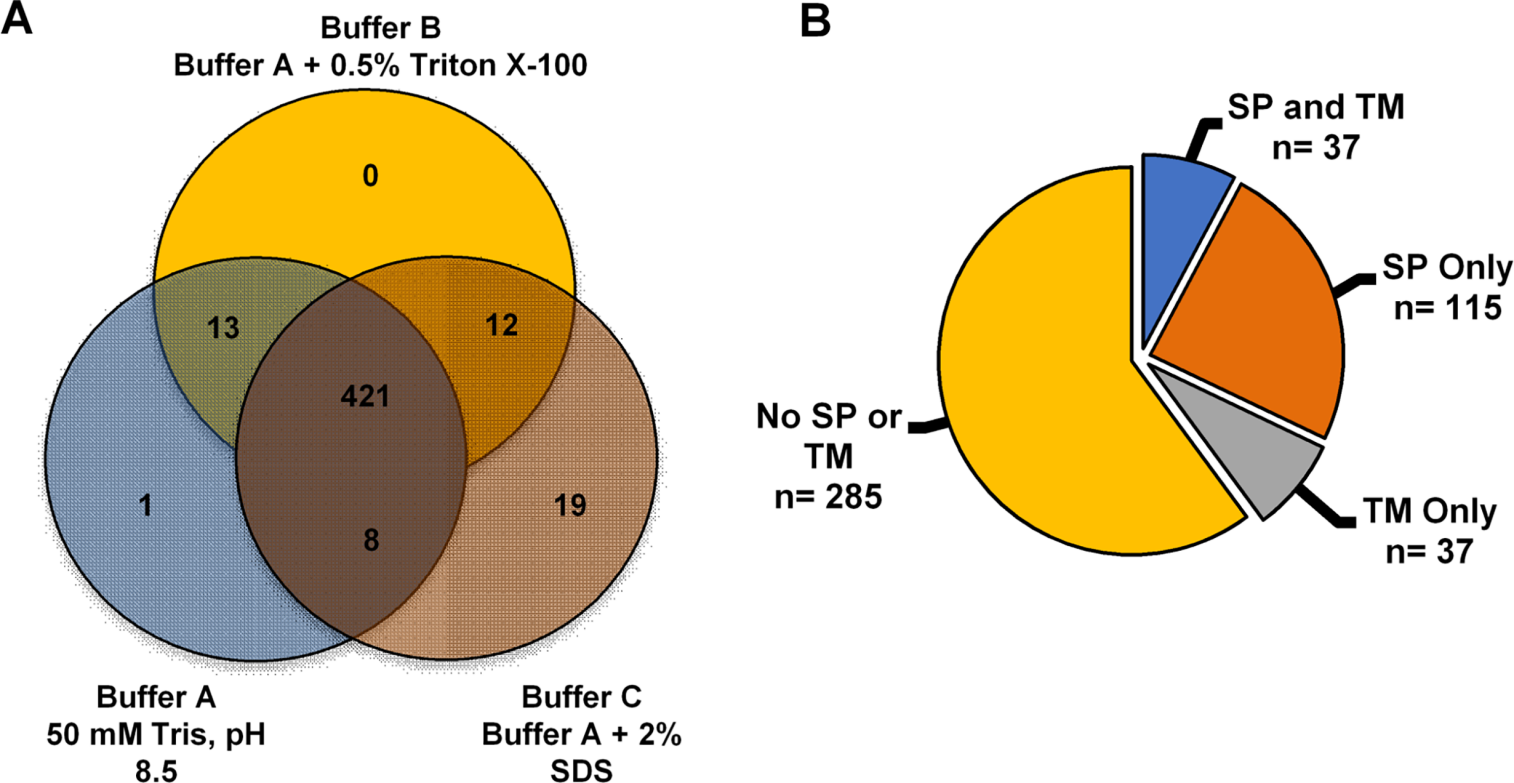
*Ae*. *aegypti* peritrophic matrix proteins by fraction and predicted localization. (**A**) Protein solubility in buffers and detergents; a total of 474 unique proteins were identified by mass spectrometry. (**B**) Categorization of peritrophic matrix proteins identified by mass spectrometry based on predicted structural features; signal peptide without transmembrane domain (SP), transmembrane domain without SP (TM), those that contain both SP and TM, and the remaining isolated proteins (No SP or TM).

Despite the stepwise extraction method, eighty-eight percent (n = 421) of the unique proteins identified were present in all three fractions (Fig 4A). Thirteen proteins were present in both fractions A and B. Eight proteins were present in both fractions A and C. Thirteen proteins were present in fractions A and B. Fourteen proteins were present in both fractions B and C. While 19 proteins were found only in fraction C, none were unique to fraction B, and only one protein was unique to fraction A (Fig 4A). Predicted structural features were used to categorize the 474 unique proteins identified by LC-MS based proteomic analysis. Of the 474 unique proteins, thirty-two percent (n = 152 proteins) contained predicted secretory signal peptides (S1 Table). Thirty-seven of the 152 proteins containing predicted signal peptides also contained predicted transmembrane domains (Fig 4B; S1 Table), and thus are potential midgut surface proteins as opposed to candidate PM proteins [33]. Of most interest were the remaining 115 proteins that contained predicted signal peptides without predicted transmembrane domains (Fig 4B; S1 Table).

### *Aedes aegypti* salivary gland proteins

One of the most striking findings was the presence of many of the best-characterized *Ae*. *aegypti* salivary proteins, including D7 (AAEL006424), which was the most abundant protein in our dataset (S1 Table). In total, 415 unique peptides were identified corresponding to salivary-specific or salivary-enriched gene products by comparing our protein list with previously described salivary gland transcriptomics data, saliva and salivary gland proteomics data [34–36]. Thus, about 10% of the protein content of the early PM is potentially salivary-derived. Specifically, we identified proteins encoded by 35/183 (19%) salivary-enriched and 14/40 (35%) of the salivary-specific transcripts described by Ribeiro et al [34]. Potentially, these proteins were transferred to the midgut during ingestion of the protein-free artificial meal and were trapped by the low-melt agarose. In addition to the D7 protein listed above, we recovered *aegyptin* (AAEL010235), D7 long1 (AAEL006417), two C-type lectins (AAEL000533 and AAEL000556), antigen-5 (AAEL003053), two apyrases (AAEL006347 and AAEL006333), and the three serpins (AAEL007420, AAEL003182, AAEL002704).

### *Aedes aegypti* blood meal-regulated genes

**Fig 5:**
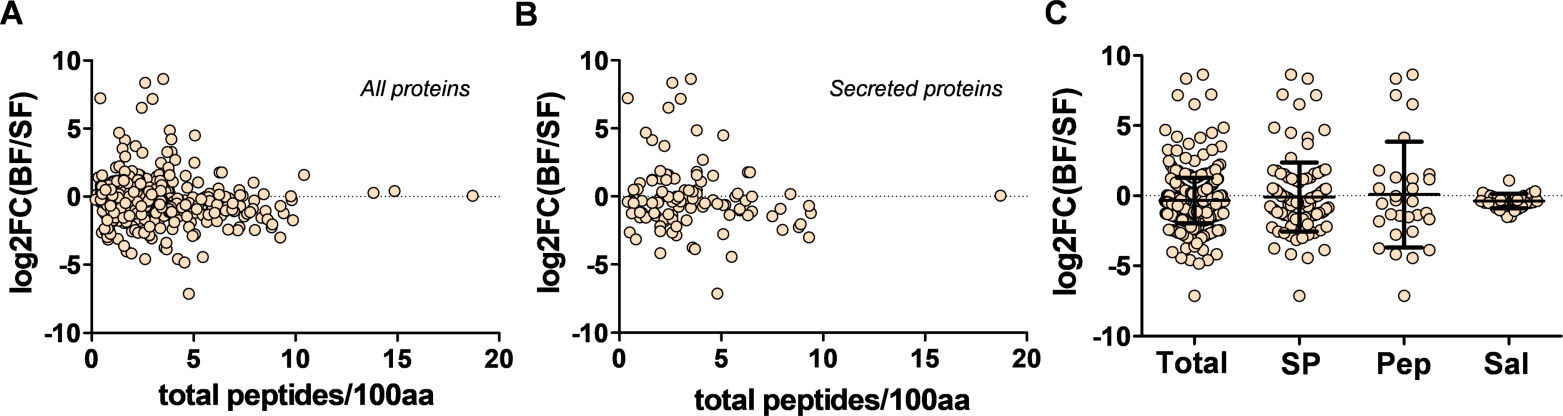
Peptide recovery from peritrophic matrix is independent of whether transcripts are regulated by a blood meal. Relationship between the size-normalized number of peptides recovered and the regulation of the corresponding transcript at 5 hours post-blood meal [30] for all identified proteins (**A**) or predicted secreted proteins (**B**). (**C**) Transcript regulation for all recovered proteins (Total), predicted secreted proteins (SP), peptidases (Pep) and salivary-enriched proteins (Sal). Error bars indicate the standard deviation from the mean.

Several RNAseq studies have been performed to identify transcripts that are differentially regulated following a blood meal in *Ae*. *aegypti* [30]. To determine whether transcripts that are differentially regulated at 5 h following a blood meal were more or less likely to result in proteins detected in our dataset, we compared the log_2_ ratio of transcript abundance between bloodfed vs. sugarfed mosquitoes from Bonizzoni et al. [30] with the length-normalized number of peptides recovered per protein for all 474 proteins in our dataset. Interestingly, there appeared to be no global relationship between the direction (up or down-regulated) or magnitude of gene expression change following a blood meal and the number of peptides that we subsequently recovered from the early PM (Fig 5A). This lack of predictive power held when considering only predicted secreted proteins (Fig 5B), peptidases or salivary proteins (Fig 5C). Likewise, the absolute abundance of transcripts from sugarfed or bloodfed mosquitoes had little to no relationship with our ability to recover the corresponding proteins (S2 Fig).

### GO term enrichment of predicted secreted proteins

**Fig 6:**
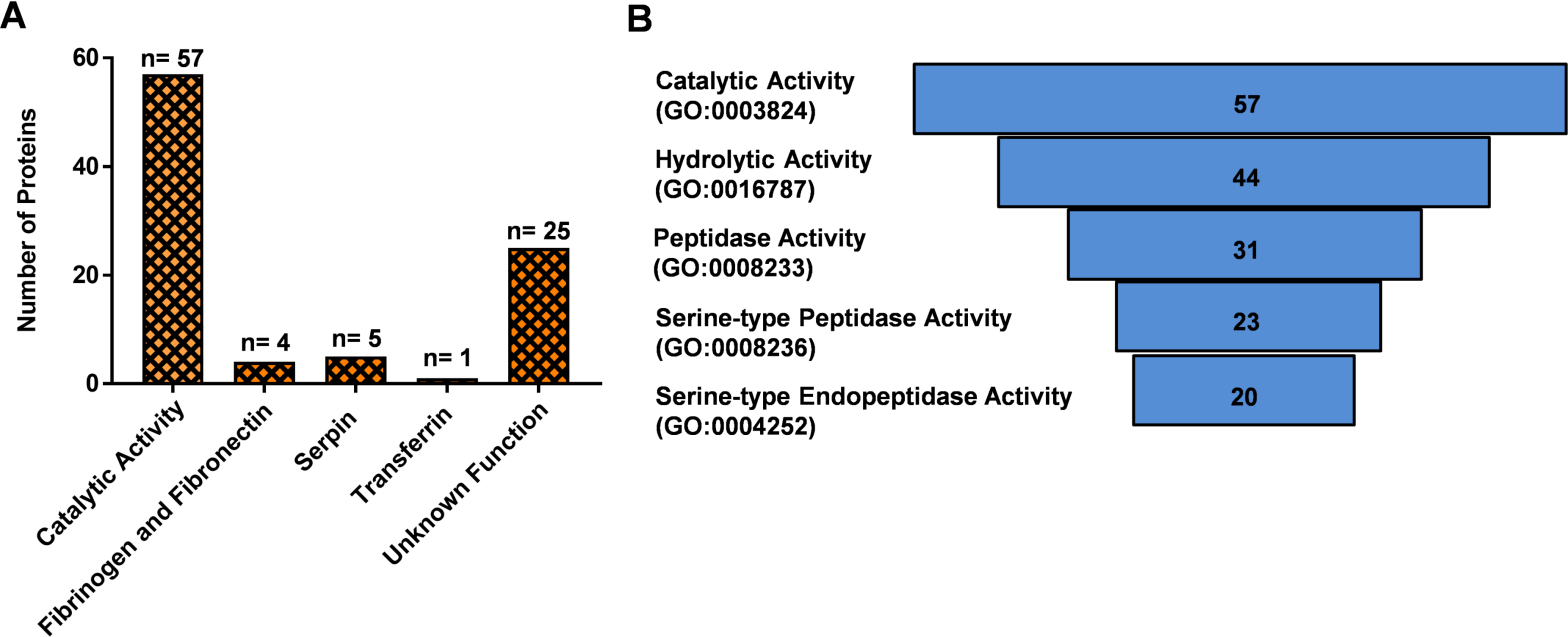
Adult *Aedes aegypti* secreted proteins isolated in this female peritrophic matric LC-MS proteomic analysis. (**A**) Bar graph detailing different groupings of the secreted proteins isolated in our proteomic analysis. (**B**) Secreted proteins with catalytic activity, hydrolase activity, peptidase activity, serine-type peptidase activity, and serine-type endopeptidase activity. Hierarchical sorting based on gProfiler g: GOSt—gene group functional profiling (p<0.001).

A gene ontology enrichment analysis for the predicted secreted proteins [31, 32] revealed that the largest number (n = 57) were associated with catalytic activity (GO:0003824) (Fig 6A and 6B). As expected, the serine-type peptidases involved in blood digestion were the largest group of secreted midgut proteins isolated in our analysis [37]. In particular, we isolated early trypsin (AaET, AAEL007818), late trypsin (AaLT, AAEL013284), serine collagenase (AAEL007432, AaSP1), and AAEL013628 (AaSP4) along with 19 other serine-type peptidases [38–40] (S1 Table).

The mosquito midgut is the first site of contact for pathogens ingested during bloodfeeding. Correspondingly, we identified a number of secreted immune-related proteins, including four fibrinogen and fibronectin proteins (AAEL004156, AAEL000726, AAEL006704, AAEL007942), two transferrins (AAEL015458, AAEL011641), five serpins (AAEL002704, AAEL002720, AAEL003182, AAEL003686 and AAEL007420), two Niemann-pick type C-2 proteins (AAEL015136 and AAEL009760), four clip-domain serine proteases (AAEL000028, AAEL006674, AAEL003625, AAEL006576), cathepsin B and L (AAEL009637 and AAEL002833), three galectins (AAEL012135, AAEL005293, AAEL009842), prophenoloxidase (AAEL013498), and lysosomal aspartic protease (AAEL006169) [30, 41–44]. Finally, six of the 115 secreted proteins without transmembrane domains isolated in our proteomic analysis were novel proteins with unknown function (S1 Table).

### Chitin-binding proteins

Only two adult female *Ae*. *aegypti* PM proteins have been identified and characterized previously, *Aedes aegypti* intestinal mucin 1 (*Ae*IMUC1) [21] and *Aedes aegypti* adult peritrophin 50 (Ae-Aper50) [22]. We identified both Ae-Aper50 (AAEL002467) and AeIMUC1 (AAEL002495), as well as a closely related gene (AAEL004798) and a fourth unrelated peritrophin gene AAEL006953 (Table 1).

**Table 1:** Known and putative adult *Ae*. *aegypti* peritrophic matrix proteins identified by LC-MS with predicted chitin-binding domains. The mass is based on the primary amino acid sequence and does not account for glycosylation.

Gene Stable ID ^a^	Gene Name	Predicted MW ^b^	Annotation/Comments ^b^^,^^c^^,^^d^
AAEL002495	AeIMUC1	30.6	3 CBD; Peritrophin-A domain; Mucin domain; O-glycosylated; SP; UF
AAEL002467	AeAper50	54.2	5 CBD; Peritrophin-A domain; N- and O-glycosylated; SP; UF
AAEL006953	-	31.3	2 CBD; Peritrophin-A domain; N-glycosylated; SP; UF
AAEL004798	-	39.6	3 CBD; Peritrophin-A domain; Mucin domain; O-glycosylated; SP; TM; UF

^a^VectorBase, *Ae*. *aegyti* mosquito database, August 2017.

^b^ The Universal Protein Resource (UniProt), August 2017.

^c^ Center for Biological Sequence Analysis (CBS) prediction services used to determine O-glycosylation and N-glycosylation status, August 2017.

^d^ CBD, chitin-binding domain; SP, signal peptide; TM, transmembrane domain; UF, unknown function.

### Orthologs to *Anopheles gambiae* PM proteomic analysis

**Fig 7:**
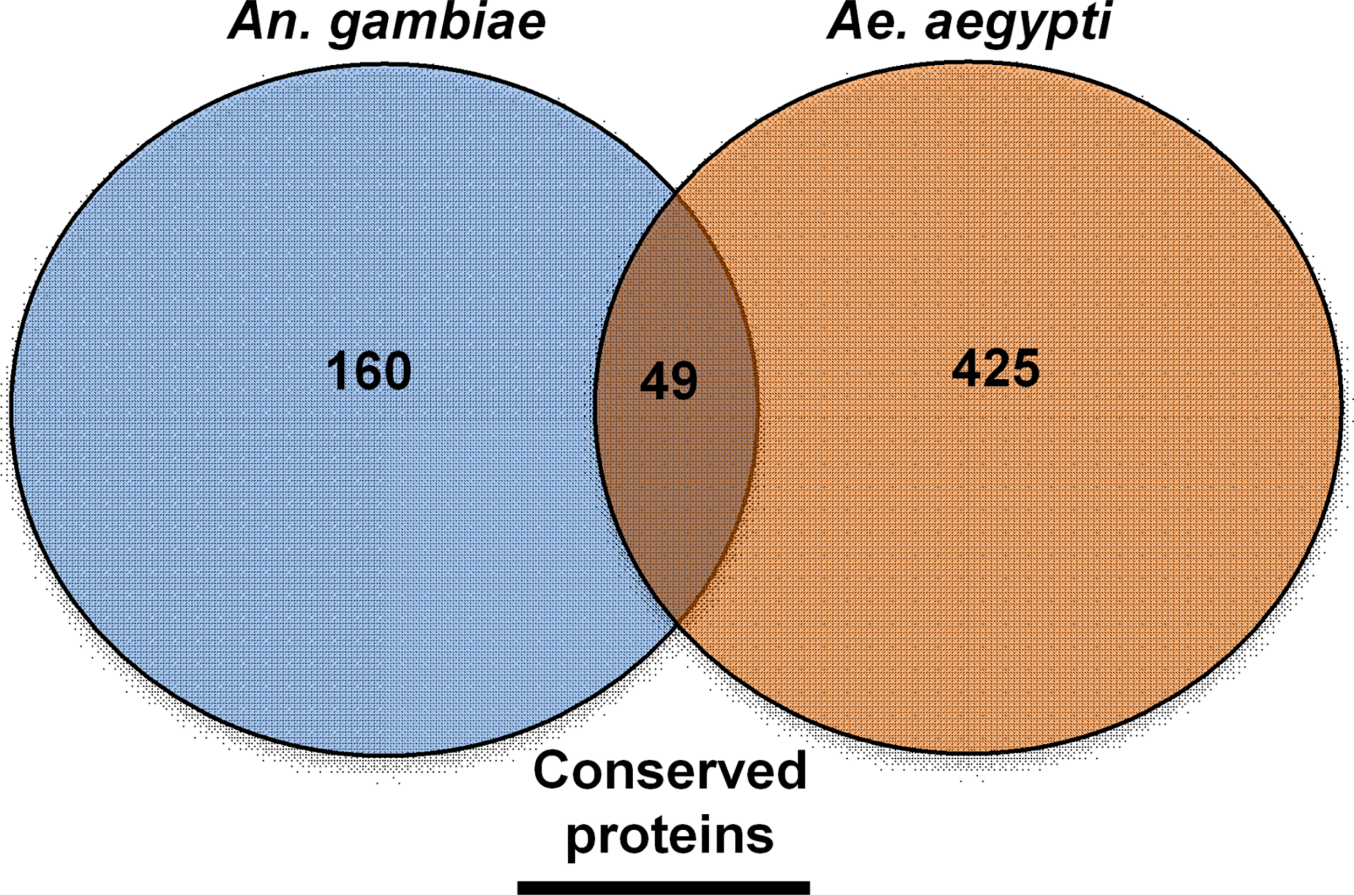
Adult *Ae*. *aegypti* peritrophic matrix proteins with *An*. *gambiae* orthologs isolated in Dinglasan et al. [33] midgut peritrophic matrix proteomic analysis.

A similar adult female PM proteomic analysis was conducted for *Anopheles gambiae* [33]. Therefore, we compared our midgut PM proteomic results to determine conserved proteins present in both Aedes and Anopheles PMs. Of the 209 unique *An*. *gambiae* PM proteins identified by Dinglasan et al. [33], 49 have one to one orthologs present in our *Ae*. *aegypti* PM proteomic analysis (Fig 7; S1 Table). More specifically, just ten were classified as 1:1 orthologs and were predicted secreted proteins without transmembrane domains (Table 2). Overall, this suggests a potentially important physiological role for these proteins, as they have been conserved in two species that diverged more than 150 million years ago [45].

**Table 2:** Adult female *Ae*. *aegypti* secreted proteins and one to one orthologs isolated in Dinglasan et al. [33] *An*. *gambiae* adult female LC-MS peritrophic matrix proteomic analysis.

*Ae*. *aegypti* Gene ID^a^	*An*. *gambiae* Gene ID^a^	Description^a^	% Identity
AAEL006347	AGAP011026	Apyrase Precursor	55.8
AAEL003066	AGAP006414	Brain chitinase and chia protein	56.6
AAEL008485	AGAP007663	DUF1397	62.0
AAEL010338	AGAP009313	DUF725	29.6
AAEL013775	AGAP007745	-	37.0
AAEL015136	AGAP002851	Niemann-Pick Type C-2, putative protein	50.3
AAEL012359	AGAP007120	Nucleoside-diphosphate kinase NBR-A, putative protein	87.5
AAEL007926	AGAP011442	Retinoid-inducible serine carboxypeptidase (serine carboxypeptidase protein	71.1
AAEL003046	AGAP001082	Saposin protein	62.1
AAEL008784	AGAP004900	Serine-type enodpeptidase, protein	60.8

^a^VectorBase, *Ae*. *aegyti* mosquito database, August 2017.

## Discussion

*Ae*. *aegypti* feed on blood multiple times during their lifespan. While critical for adult reproduction, this leaves the midgut, a tissue not protected by chitinous cuticle, exposed to potential toxins, pathogens, and other abrasive compounds during blood digestion [14, 16, 46]. It has been hypothesized that the peritrophic matrix- an acellular sheath comprised of proteins, proteoglycans and chitin fibrils, may serve as a protective lining that separates the single cell-layered midgut epithelium from these hazards [7, 8, 14, 15]. Therefore, we conducted a mass spectrometry based proteomic analysis to obtain a comprehensive understanding of the adult female *Ae*. *aegypti* PM. A protein-based analysis of a tissue such as the PM, which is normally induced following a blood meal, is complicated by the large excess of protein present in the blood. Dinglasan et al. [33] overcame this in *An*. *gambiae* by performing an artificial feed using a protein-free solution containing latex beads to aid in midgut distension. Our substitution of low-melt agarose into the protein-free solution simplified the dissection procedure, as once solidified the agarose provided internal rigidity to an otherwise fragile structure. Fortuitously, this also allowed the capture of both structural components of the PM as well as soluble proteins secreted and trapped in the lumen of the midgut. Based on two-dimensional polyacrylamide gel electrophoresis, Moskalyk et al. [20] determined that the adult female *Ae*. *aegypti* PM may contain 20–40 major proteins [20]. In contrast, our LC-MS based proteomic analysis resulted in the identification of 474 unique proteins. Computational resampling indicated this is very close to saturation, suggesting we have achieved a substantially complete (96–98%) view of the early PM. At the same time, it is possible that some proteins were missed due to extensive glycosylation or other post-translational modifications that no peptides could be recognized. In addition, we cannot rule out that some components of the PM are selectively produced only following an authentic blood meal, but not from our protein-free meal. However, we consider this unlikely to substantially reduce the completeness of our dataset for several reasons. First, the PM is known to form rapidly based solely on distension of the midgut [14, 47]. Second, we identified a large number of digestive enzymes including early trypsin known to be secreted into the midgut lumen and responsible for a preliminary tasting of the meal [37–40, 48–50]. Third, we identified both previously characterized *Ae*. *aegypti* PM peritrophins, AeIMUC1 [21] and AeAper50 [22].

In addition to chitin, peritrophins have also been shown to bind blood meal derived heme [8]. Pascoa et al. [8] demonstrated the heme binding capacity of the adult *Ae*. *aegypti* PM. They found that by the end of digestion the adult *Ae*. *aegypti* PM could bind 18 nmol of heme, which is an equivalent to the amount of heme present in a normal blood meal. AeIMUC1 is a 275-amino acid glycoprotein with a 19-amino acid secretory signal peptide sequence [7, 21] and contains three chitin-binding domains and a mucin domain between CBD 1 and CBD 2. Rayms-Keller et al. [21] first reported *Ae*IMUC1 RNA expression in metal exposed *Ae*. *aegypti* mosquito larvae, metal fed adult females and blood-fed adult females [21]. Devenport et al. [7], found that AeIMUC1 could bind chitin and heme, suggesting a role in blood meal detoxification. Through deletion analysis, Devenport et al [7] also determined that the heme-binding activity of AaIMUC1 was associated with its 3 CBDs. Finally, AeIMUC1 was confirmed as an integral PM peritrophin associated with the PM 12 to 24 hours post bloodfeeding, and that this protein is translationally regulated by bloodfeeding [7].

AeAper50 is a 486-amino acid protein that contains 18-amino acid secretory signal peptide [22]. AeAper50 is localized in the midgut of blood-fed adult females, and the protein is present within just one hour of adult female feeding [22]. In contrast to AeIMUC1 [7], mRNA for AeAper50 was not shown to be present prior to adult female bloodfeeding, but rapidly accumulated after the blood meal [22]. Shao et al. [22] also confirmed chitin-binding for AeAper50. More specifically, through site-directed mutagenesis (cysteine to alanine) of AeAper50 CBD 5, Shao et al. [22] demonstrated the importance of peritrophin-A domain (PAD) (six cysteine residues) conserved cysteine residues for disulfide bridge formation. These results suggest that the disulfide bridges position AeAper50 for chitin fibril binding [17, 22, 51].

We identified two additional putative PM peritrophins with structural features similar to known peritrophins: AAEL006953 is predicted to encode a 31.4 kDa protein with two predicted chitin-binding domains and N-glycosylation, while AAEL004798 is predicted to encode a 39.6 kDa protein with three predicted chitin-binding domains, a mucin domain and O-glycosylation. Additional work is needed to confirm if these *Ae*. *aegypti* PM peritrophins also bind chitin and/or heme, as RNAi mediated knockdown of *Anopheles gambiae* adult peritrophin 1 (AgAper1) resulted in bacterial proliferation and a corresponding immune response suggesting the heme-binding function of the PM may both protect the midgut from toxicity as well as sequester the heme to prevent microbial overproliferation [52]. The presence of multiple peritrophins in the PM complicates reverse genetic approaches such as RNAi, but ultimately such studies will be essential to clarifying the role of the four *Ae*. *aegypti* PM peritrophins in blood digestion, heme sequestration and immunity.

Although salivary proteins are primarily thought of in terms of blood meal acquisition, 10% of the PM protein content appears to be salivary-derived and we identified a substantial number of secreted salivary proteins associated with the PM, with the salivary D7 proteins being the most abundant protein when normalized by length (most peptides per 100 amino acids of protein). The simplest explanation is the ingestion of salivary proteins during feeding; Anopheline mosquitoes have been known to ingest malaria parasites originating from their salivary glands during the act of bloodfeeding [53, 54]. However, we cannot exclude the possibility that some of these proteins may also be produced to some extent by the midgut, as other PM proteomic studies have also isolated proteins traditionally ascribed to the salivary glands and saliva [55]. While several early studies found that severing of the salivary ducts of *Ae*. *aegypti* had little to no effect on blood meal digestion and subsequent egg production [56, 57], the difficulty and low survivorship associated with these microsurgeries resulted in extremely low sample sizes and thus limited the statistical power of these data. Genetic lesions in important salivary protein genes made possible now through Cas9-based gene editing will allow further investigation as to the physiological importance of salivary proteins and their relationship to the PM, blood digestion and mosquito reproduction.

Similar to our findings, Dinglasan et al. [33] also isolated salivary-associated proteins [33]. Interestingly, the authors isolated *An*. *gambiae* apyrase (Table 2), an enzyme known to inhibit ADP-dependent platelet aggregation [58] and whose expression is specific to secretory cells of the distal-lateral lobes of adult female *Ae*. *aegypti* [59]. Given, that previous studies have also shown large amounts of saliva are ingested during feeding in hematophagous insects [57, 60], these salivary-associated proteins may have been trapped in the lumen when ingested, as our artificial meal included low melting agarose. However, further studies are needed to determine if apyrase is also functional in midgut blood meal digestion across hematophagous arthropods as it was one of the ten proteins found associated with the PM in both *An*. *gambiae* and *Ae*. *aegypti*.

As the first comprehensive proteomic analysis for the adult female *Ae*. *aegypti* midgut PM, our findings provide a foundation for future studies which are needed to better understand the physiological role of the PM after adult female bloodfeeding. While further strides have been taken to determine physiological function and importance of the PM in other hematophagous arthropods [52, 55, 61], reverse genetic analyses (RNAi mediated knockdown) are needed to confirm physiological function for known and putative *Ae*. *aegypti* PM proteins [61]. Likewise, *in vitro* heme-binding assays are needed to confirm heme-binding for known and putative PM proteins. Furthermore, an enrichment proteomic analysis comparing our current artificial meal to that of an artificial meal enriched with heme provides the ideal avenue for identifying additional PM heme-binding proteins, which were not isolated in our current proteomic analysis. Overall, understanding the PM and its components has the potential to provide novel targets for molecular based vector and vector-borne disease control, as well as understanding the adaptations required for efficient blood digestion/detoxification.

## Supporting information

S1 Fig Peptide length distribution. Frequency distribution of peptide length versus abundance for all 6319 peptides obtained from *Ae*. *aegypti* peritrophic matrix and contents.
(TIF)

S2 Fig Peptide recovery from peritrophic matrix is only weakly correlated with transcript abundance. Relationship between the length-normalized transcript abundance in bloodfed (**A**) or sugar-fed (**B**) mosquitoes.
(TIF)

S1 Table *Aedes aegypti* early peritrophic matrix proteome full dataset. Master counts (Tab 1) for 474 PM-associated proteins. Column headings include: Vectorbase gene ID (AaegL3.3), Interpro description, Interpro ID, GO terms, isoelectic point (IEP), predicted molecular weight (mw), max score, protein ID, # unique peptides, # total peptides, % sequence coverage, false discovery rate level (FDR), predicted cleavage site (SignalP), Total intensity and intensity in extracts A, B and C, bloodfed (BF) and sugar-fed (SF) mRNAseq counts from Bonizonni et al (2011). Table also includes raw peptide data (Peptide IDs; Tab 2), identified proteins per fraction (Tab 3), as well as subsets of predicted secreted proteins (Tab 3), salivary proteins (Tab 4) and *An*. *gambiae* 1:1 orthologs (Tab 5).
(XLSX)

## References

[1] Black IV WC, Bennett KE, Gorrochótegui-Escalante N, Barillas-Mury CV, Fernández-Salas I, de Lourdes Muñoz Ma, et al Flavivirus Susceptibility in *Aedes aegypti*. Archives of Medical Research. 2002;33(4):379–88. 12234528 10.1016/s0188-4409(02)00373-9

[2] Gubler DJ, Clark GG. Dengue/dengue hemorrhagic fever: the emergence of a global health problem. Emerging Infectious Diseases. 1995;1(2):55–7. doi: 10.3201/eid0102.952004 8903160 10.3201/eid0102.952004 PMC2626838

[3] Rothman AL, Ennis FA. Immunopathogenesis of Dengue Hemorrhagic Fever. Virology. 1999;257(1):1–6. doi: 10.1006/viro.1999.9656 10208914 10.1006/viro.1999.9656

[4] Murray NEA, Quam MB, Wilder-Smith A. Epidemiology of dengue: past, present and future prospects. Clinical Epidemiology. 2013;5:299–309. doi: 10.2147/CLEP.S34440 23990732 10.2147/CLEP.S34440 PMC3753061

[5] Wilder-Smith A, Gubler DJ. Dengue vaccines at a crossroad. Science. 2015;350(6261):626–7. doi: 10.1126/science.aab4047 26542552 10.1126/science.aab4047

[6] Oliveira JHM, Gonçalves RLS, Lara FA, Dias FA, Gandara ACP, Menna-Barreto RFS, et al Blood Meal-Derived Heme Decreases ROS Levels in the Midgut of *Aedes aegypti* and Allows Proliferation of Intestinal Microbiota. PLoS Pathog. 2011;7(3):e1001320 doi: 10.1371/journal.ppat.1001320 21445237 10.1371/journal.ppat.1001320 PMC3060171

[7] Devenport M, Alvarenga PH, Shao L, Fujioka H, Bianconi ML, Oliveira PL, et al Identification of the *Aedes aegypti* Peritrophic Matrix Protein AeIMUCI as a Heme-Binding Protein†. Biochemistry. 2006;45(31):9540–9. doi: 10.1021/bi0605991 16878988 10.1021/bi0605991

[8] Pascoa V, Oliveira PL, Dansa-Petretski Ml, Silva JR, Alvarenga PH, Jacobs-Lorena M, et al *Aedes aegypti* peritrophic matrix and its interaction with heme during blood digestion. Insect Biochemistry and Molecular Biology. 2002;32(5):517–23. 11891128 10.1016/s0965-1748(01)00130-8

[9] Ryter SW, Tyrrell RM. The heme synthesis and degradation pathways: role in oxidant sensitivity: Heme oxygenase has both pro- and antioxidant properties. Free Radical Biology and Medicine. 2000;28(2):289–309. 11281297 10.1016/s0891-5849(99)00223-3

[10] Tappel AL. Unsaturated lipid oxidation catalyzed by hematin compounds. Journal of Biological Chemistry. 1955;217:721–33. 13271434

[11] Vincent SH, Grady RW, Shaklai N, Snider JM, Muller-Eberhard U. The influence of heme-binding proteins in heme-catalyzed oxidations. Archives of biochemistry and biophysics. 1988;265(2):539–50. 3421724 10.1016/0003-9861(88)90159-2

[12] Aft RL, Mueller GC. Hemin-mediated oxidative degradation of proteins. Journal of Biological Chemistry. 1984;259(1):301–5. 6323403

[13] Vincent S, editor Oxidative effects of heme and porphyrins on proteins and lipids. Seminars in hematology; 1989. 2658086

[14] Richards AG, Richards PA. The Peritrophic Membranes of Insects. Annual Review of Entomology. 1977;22(1):219–40. 10.1146/annurev.en.22.010177.001251 319739

[15] Stohler H. The peritrophic membrane of blood-sucking Diptera in relation to their role as vectors of blood parasites. Acta Tropica. 1961;18:263–6.

[16] Shao L, Devenport M, Jacobs-Lorena M. The peritrophic matrix of hematophagous insects. Archives of Insect Biochemistry and Physiology. 2001;47(2):119–25. doi: 10.1002/arch.1042 11376458 10.1002/arch.1042

[17] Tellam RL, Wijffels G, Willadsen P. Peritrophic matrix proteins. Insect Biochemistry and Molecular Biology. 1999;29(2):87–101. 10196732 10.1016/s0965-1748(98)00123-4

[18] Shen Z, Jacobs-Lorena M. A Type I Peritrophic Matrix Protein from the Malaria Vector *Anopheles gambiae* Binds to Chitin: Cloning, Expression, and Characterization. Journal of Biological Chemistry. 1998;273(28):17665–70. 9651363 10.1074/jbc.273.28.17665

[19] Schorderet S, Pearson RD, Vuocolo T, Eisemann C, Riding GA, Tellam RL. cDNA and deduced amino acid sequences of a peritrophic membrane glycoprotein, `Peritrophin-48', from the larvae of *Lucilia cuprina*. Insect Biochemistry and Molecular Biology. 1998;28(2):99–111. 9639876 10.1016/s0965-1748(97)00103-3

[20] Moskalyk LA, Oo MM, Jacobs-Lorena M. Peritrophic matrix proteins of *Anopheles gambiae* and *Aedes aegypti*. Insect Molecular Biology. 1996;5(4):261–8. 8933177 10.1111/j.1365-2583.1996.tb00100.x

[21] Rayms-Keller A, McGaw M, Oray C, Carlson JO, Beaty BJ. Molecular cloning and characterization of a metal responsive *Aedes aegypti* intestinal mucin cDNA. Insect Molecular Biology. 2000;9(4):419–26. 10971719 10.1046/j.1365-2583.2000.00202.x

[22] Shao L, Devenport M, Fujioka H, Ghosh A, Jacobs-Lorena M. Identification and characterization of a novel peritrophic matrix protein, Ae-Aper50, and the microvillar membrane protein, AEG12, from the mosquito, *Aedes aegypti*. Insect Biochemistry and Molecular Biology. 2005;35(9):947–59. doi: 10.1016/j.ibmb.2005.03.012 15978997 10.1016/j.ibmb.2005.03.012

[23] Kogan PH. Substitute Blood Meal for Investigating and Maintaining *Aedes aegypti* (Diptera: Culicidae). Journal of Medical Entomology. 1990;27(4):709–12. 2388248 10.1093/jmedent/27.4.709

[24] Galun R, Avi-Dor Y, Bar-Zeev M. Feeding Response in *Aedes aegypti*: Stimulation by Adenosine Triphosphate. Science. 1963;142(3600):1674–5. doi: 10.1126/science.142.3600.1674 17834375 10.1126/science.142.3600.1674

[25] Marquardt WC, Kondratieff BC. Biology of disease vectors 2nd ed Burlington, MA: Elsevier Academic Press; 2005 xxiii, 785 p. p.

[26] Wolters DA, Washburn MP, Yates JR. An Automated Multidimensional Protein Identification Technology for Shotgun Proteomics. Analytical Chemistry. 2001;73(23):5683–90. 11774908 10.1021/ac010617e

[27] Distler U, Kuharev J, Navarro P, Levin Y, Schild H, Tenzer S. Drift time-specific collision energies enable deep-coverage data-independent acquisition proteomics. Nat Meth. 2014;11(2):167–70. 10.1038/nmeth.2767 24336358

[28] Liao Z, Wan Y, Thomas SN, Yang AJ. IsoQuant: A Software Tool for SILAC-Based Mass Spectrometry Quantitation. Analytical chemistry. 2012;84(10):4535–43. doi: 10.1021/ac300510t 22519468 10.1021/ac300510t PMC3583527

[29] Vizcaíno Juan A, Csordas A, del-Toro N, Dianes JA, Griss J, Lavidas I, et al 2016 update of the PRIDE database and its related tools. Nucleic Acids Research. 2016;44(D1):D447–D456. doi: 10.1093/nar/gkv1145 26527722 10.1093/nar/gkv1145 PMC4702828

[30] Bonizzoni M, Dunn WA, Campbell CL, Olson KE, Dimon MT, Marinotti O, et al RNA-seq analyses of blood-induced changes in gene expression in the mosquito vector species, *Aedes aegypti*. BMC Genomics. 2011;12(1):82. 21276245 10.1186/1471-2164-12-82 PMC3042412

[31] Reimand J, Kull M, Peterson H, Hansen J, Vilo J. g:Profiler—a web-based toolset for functional profiling of gene lists from large-scale experiments. Nucleic Acids Research. 2007;35(suppl 2):W193–W200. 17478515 10.1093/nar/gkm226 PMC1933153

[32] Reimand J, Arak T, Vilo J. g:Profiler—a web server for functional interpretation of gene lists (2011 update). Nucleic Acids Research. 2011;39(suppl 2):W307–W15. 21646343 10.1093/nar/gkr378 PMC3125778

[33] Dinglasan RR, Devenport M, Florens L, Johnson JR, McHugh CA, Donnelly-Doman M, et al The *Anopheles gambiae* adult midgut peritrophic matrix proteome. Insect Biochemistry and Molecular Biology. 2009;39(2):125–34. doi: 10.1016/j.ibmb.2008.10.010 19038338 10.1016/j.ibmb.2008.10.010 PMC2684889

[34] Ribeiro JMC, Martin-Martin I, Arcà B, Calvo E. A Deep Insight into the Sialome of Male and Female *Aedes aegypti* Mosquitoes. PLoS ONE. 2016;11(3):e0151400 doi: 10.1371/journal.pone.0151400 26999592 10.1371/journal.pone.0151400 PMC4801386

[35] Conway MJ, Londono-Renteria B, Troupin A, Watson AM, Klimstra WB, Fikrig E, et al *Aedes aegypti* D7 Saliva Protein Inhibits Dengue Virus Infection. PLoS Negl Trop Dis. 2016;10(9):e0004941 doi: 10.1371/journal.pntd.0004941 27632170 10.1371/journal.pntd.0004941 PMC5025043

[36] Ribeiro JMC, Arcà B, Lombardo F, Calvo E, Van My P, Chandra PK, et al An annotated catalogue of salivary gland transcripts in the adult female mosquito, *Aedes ægypti*. BMC Genomics. 2007;8:6–. doi: 10.1186/1471-2164-8-6 17204158 10.1186/1471-2164-8-6 PMC1790711

[37] Brackney DE, Isoe J, W.C. Iv B, Zamora J, Foy BD, Miesfeld RL, et al Expression profiling and comparative analyses of seven midgut serine proteases from the yellow fever mosquito, *Aedes aegypti*. Journal of Insect Physiology. 2010;56(7):736–44. doi: 10.1016/j.jinsphys.2010.01.003 20100490 10.1016/j.jinsphys.2010.01.003 PMC2878907

[38] Noriega FG, Pennington JE, Barillas-Mury C, Wang XY, Wells MA. *Aedes aegypti* midgut early trypsin is post-transcriptionally regulated by blood feeding. Insect Molecular Biology. 1996;5(1):25–9. 8630532 10.1111/j.1365-2583.1996.tb00037.x

[39] Barillas-Mury CV, Noriega FG, Wells MA. Early trypsin activity is part of the signal transduction system that activates transcription of the late trypsin gene in the midgut of the mosquito, *Aedes aegypti*. Insect Biochemistry and Molecular Biology. 1995;25(2):241–6. 7711754 10.1016/0965-1748(94)00061-l

[40] Noriega FG, Wells MA. A molecular view of trypsin synthesis in the midgut of *Aedes aegypti*. Journal of Insect Physiology. 1999;45(7):613–20. 12770346 10.1016/s0022-1910(99)00052-9

[41] Lowenberger C. Innate immune response of *Aedes aegypti*. Insect Biochemistry and Molecular Biology. 2001;31(3):219–29. 11167091 10.1016/s0965-1748(00)00141-7

[42] Hanington PC, Zhang SM. The Primary Role of Fibrinogen-Related Proteins in Invertebrates Is Defense, Not Coagulation. Journal of Innate Immunity. 2011;3(1):17–27. doi: 10.1159/000321882 21063081 10.1159/000321882 PMC3031514

[43] Conus S, Simon H-U. Cathepsins and their involvement in immune responses. Swiss Med Wkly. 2010;140(w13042). 10.4414/smw.2010.13042 20648403

[44] Matsumoto F, Saitoh S-i, Fukui R, Kobayashi T, Tanimura N, Konno K, et al Cathepsins are required for Toll-like receptor 9 responses. Biochemical and Biophysical Research Communications. 2008;367(3):693–9. doi: 10.1016/j.bbrc.2007.12.130 18166152 10.1016/j.bbrc.2007.12.130

[45] Krzywinski J, Grushko OG, Besansky NJ. Analysis of the complete mitochondrial DNA from *Anopheles funestus*: An improved dipteran mitochondrial genome annotation and a temporal dimension of mosquito evolution. Molecular Phylogenetics and Evolution. 2006;39(2):417–23. doi: 10.1016/j.ympev.2006.01.006 16473530 10.1016/j.ympev.2006.01.006

[46] Peters W. Peritrophic Membranes. Berlin: Springer-Verlag; 1992.

[47] Freyvogel TA, Jaquet C. The prerequisites for the formation of a peritrophic membrane in Culicidae females. Acta Tropica. 1965;22:148–54. 14319772

[48] Barillas-Mury C, Wells MA. Cloning and sequencing of the blood meal-induced late trypsin gene from the mosquito *Aedes aegypti* and characterization of the upstream regulatory region. Insect Molecular Biology. 1993;2(1):7–12. 9087537 10.1111/j.1365-2583.1993.tb00119.x

[49] Edwards MJ, Moskalyk LA, Donelly-Doman M, Vlaskova M, Noriega FG, Walker VK, et al Characterization of a carboxypeptidase A gene from the mosquito, *Aedes aegypti*. Insect Molecular Biology. 2000;9(1):33–8. 10672069 10.1046/j.1365-2583.2000.00159.x

[50] Jiang Q, Hall M, Noriega FG, Wells M. cDNA cloning and pattern of expression of an adult, female-specific chymotrypsin from *Aedes aegypti* midgut. Insect Biochemistry and Molecular Biology. 1997;27(4):283–9. 9134710 10.1016/s0965-1748(97)00001-5

[51] Toprak U, Erlandson M, Hegedus D. Peritrophic matrix proteins. Trends in Entomology. 2010;6:23–51.

[52] Rodgers FH, Gendrin M, Wyer CAS, Christophides GK. Microbiota-induced peritrophic matrix regulates midgut homeostasis and prevents systemic infection of malaria vector mosquitoes. PLOS Pathogens. 2017;13(5):e1006391 doi: 10.1371/journal.ppat.1006391 28545061 10.1371/journal.ppat.1006391 PMC5448818

[53] Yorke W, Macfie J. The action of the salivary secretion of mosquitoes and of *Glossina tachinoides* on human blood. Ann Trop Med Parasitol. 1924;18:103–8.

[54] Beier MS, Davis JR, Pumpuni CB, Noden BH, Beier JC. Ingestion of *Plasmodium falciparum* sporozoites during transmission by Anopheline mosquitoes. Am J Trop Med Hyg. 1992;47(2):195–200. 1503188 10.4269/ajtmh.1992.47.195

[55] Rose C, Belmonte R, Armstrong SD, Molyneux G, Haines LR, Lehane MJ, et al An Investigation into the Protein Composition of the Teneral *Glossina morsitans morsitans* Peritrophic Matrix. PLoS Neglected Tropical Diseases. 2014;8(4):e2691 doi: 10.1371/journal.pntd.0002691 24763256 10.1371/journal.pntd.0002691 PMC3998921

[56] Hudson A. Some Functions of Salivary Glands of Mosquitoes and Other Blood-feeding Insects. Canadian Journal of Zoology. 1964;42(1):113–120.

[57] Mellink JJ, Vandenbovenkamp W. Functional-aspects of Mosquito Salivation in Blood Feeding of *Aedes aegypti*. Mosquito News. 1981;41(1):115–9.

[58] Champagne DE, Smartt CT, Ribeiro JM, James AA. The salivary gland-specific apyrase of the mosquito *Aedes aegypti* is a member of the 5'-nucleotidase family. Proceedings of the National Academy of Sciences of the United States of America. 1995;92(3):694–8. 7846038 10.1073/pnas.92.3.694 PMC42686

[59] Juhn J, Naeem-Ullah U, Maciel Guedes BA, Majid A, Coleman J, Paolucci Pimenta PF, et al Spatial mapping of gene expression in the salivary glands of the dengue vector mosquito, *Aedes aegypti*. Parasites & Vectors. 2011;4:1–. 21205315 10.1186/1756-3305-4-1 PMC3043528

[60] Hawkins RI. Factors affecting Blood Clotting from Salivary Glands and Crop of *Glossina austeni*. Nature. 1966;212:738–9.

[61] Whiten SR, Eggleston H, Adelman ZN. Ironing out the Details: Exploring the Role of Iron and Heme in Blood-Sucking Arthropods. Frontiers in Physiology. 2018;8(1134). 10.3389/fphys.2017.01134 PMC5776124 29387018

