# Supplementary figures and images for: Characterization of the adult Aedes aegypti early midgut peritrophic matrix proteome using LC-MS
Source: PLoS One. 2018 Mar 23;13(3):e0194734. doi: 10.1371/journal.pone.0194734 (PMC5865745; doi:10.1371/journal.pone.0194734)

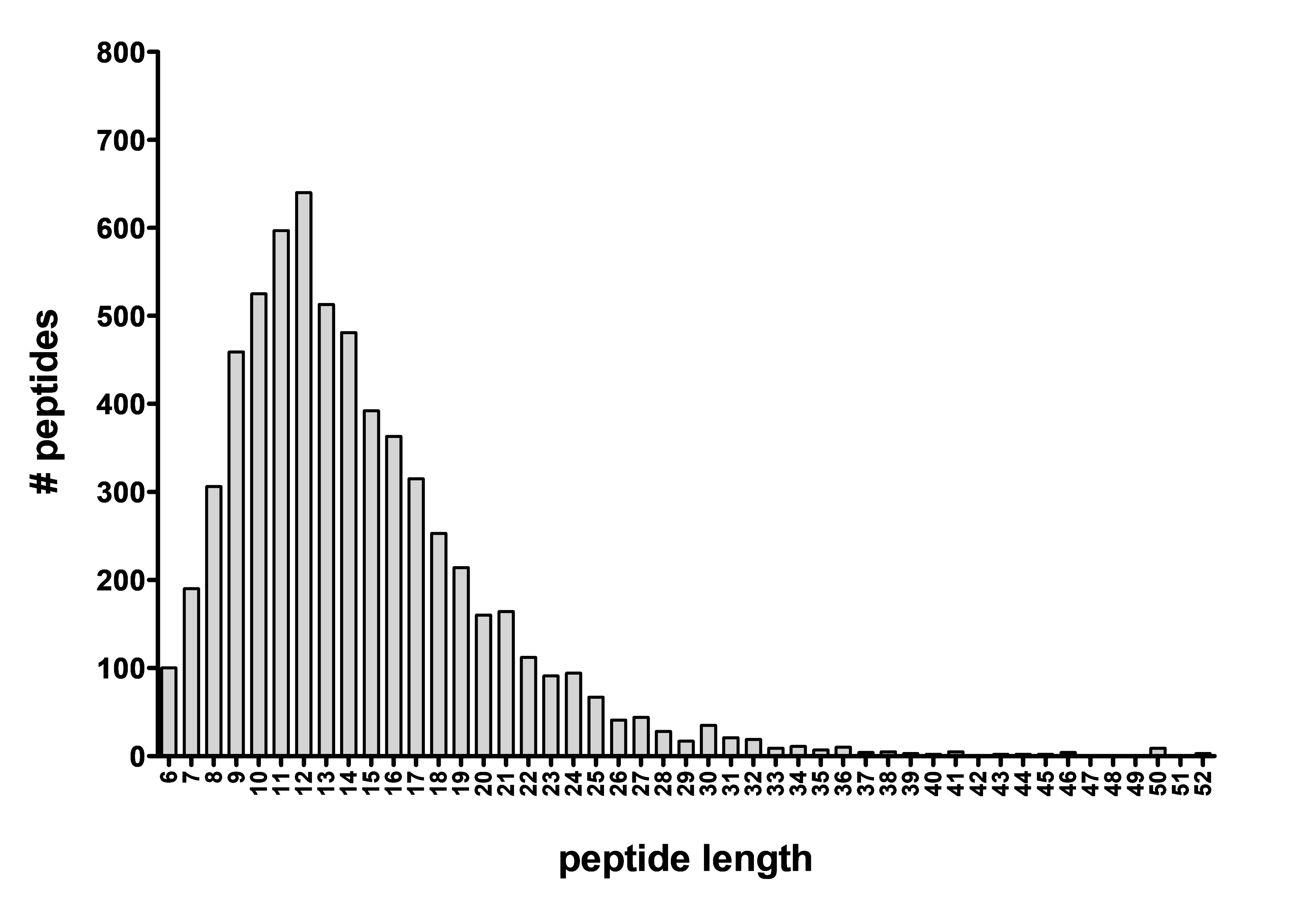

Supplement: S1 Fig — Frequency distribution of peptide length versus abundance for all 6319 peptides obtained from Ae. aegypti peritrophic matrix and contents. (TIF) [file pone.0194734.s001.tif]

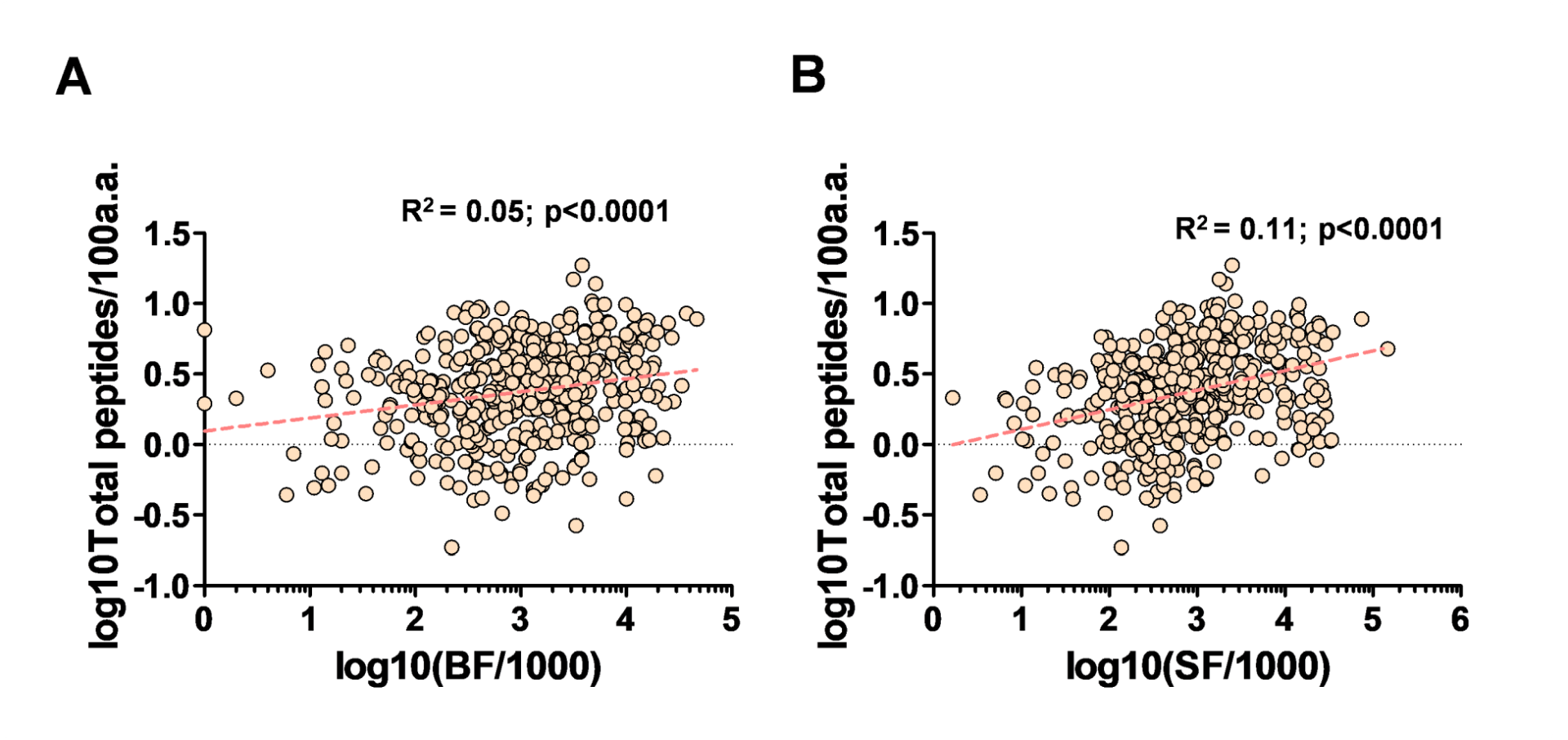

Supplement: S2 Fig — Relationship between the length-normalized transcript abundance in bloodfed (A) or sugar-fed (B) mosquitoes. (TIF) [file pone.0194734.s002.tif]
